# Aqueous Extract of Red Deer Antler Promotes Hair Growth by Regulating the Hair Cycle and Cell Proliferation in Hair Follicles

**DOI:** 10.1155/2014/878162

**Published:** 2014-02-13

**Authors:** Jing-jie Li, Zheng Li, Li-juan Gu, Yun-bo Wang, Mi-ra Lee, Chang-keun Sung

**Affiliations:** Department of Food Science and Technology, College of Agriculture and Biotechnology, Chungnam National University, 220 Gung-dong, Yusung-gu, Daejeon 305-764, Republic of Korea

## Abstract

Deer antlers are the only mammalian appendage capable of regeneration. We aimed to investigate the effect of red deer antler extract in regulating hair growth, using a mouse model. The backs of male mice were shaved at eight weeks of age. Crude aqueous extracts of deer antler were prepared at either 4°C or 100°C and injected subcutaneously to two separate groups of mice (*n* = 9) at 1 mL/day for 10 consecutive days, with water as a vehicle control group. The mice skin quantitative hair growth parameters were measured and 5-bromo-2-deoxyuridine was used to identify label-retaining cells. We found that, in both the 4°C and the 100°C deer antler aqueous extract-injection groups, the anagen phase was extended, while the number of BrdU-incorporated cells was dramatically increased. These results indicate that deer antler aqueous extract promotes hair growth by extending the anagen phase and regulating cell proliferation in the hair follicle region.

## 1. Introduction

Deer antlers are the only bony structures in mammals that completely regenerate every year, and velvet is the epidermis covering the inner structure of the growing bone and cartilage, which develops into antler. Each antler grows from a junction point on the skull called the pedicle [1]. Deer antler has a very long history of use in traditional Chinese medicine (TCM). Traditional medical reports and clinical observations suggest that antler velvet is composed of many components, such as chondroitin sulfate, estrone, estradiol, prostaglandins [2], 416 unique proteins such as vimentin [3], and growth factors including insulin-like growth factor-1 [4], and epidermal growth factor [5]. The most important components are insulin-like growth factors (IGFs) that we detected in our previous research [6]. These growth factors increase the rate of cell division, indicating a possible role in cell regeneration and repair processes in humans [7]. In addition, the proliferation effect of deer antler is studied recently [8, 9]. Previously, our research group demonstrated the beneficial effect of deer antler on wound healing in a rat model [10]. In the same study, we coincidentally observed that deer antler extract seemed to have a stimulatory effect on hair regrowth.

To produce new hairs, existing follicles undergo a process which has been defined for the mammalian hair cycle and consists of three phases: anagen, catagen, and telogen [11]. The duration of anagen determines the length of the hair and is dependent upon continued proliferation and differentiation of matrix cells at the follicle base. As the supply of matrix cells declines, differentiation of hair shaft (HS) and inner root sheath (IRS) slow down, and the follicle enters a destructive phase called catagen [12]. Following catagen, follicles lie dormant in a resting phase (telogen). Although no new hair follicles are generated postnatal, the lower portion of the hair follicle regenerates in order to produce a new hair.

Based on previous studies investigating the effect of deer antler on wound healing [10], we found that deer antler extract accelerated hair growth by enhanced IGF-1 expression in wound healing skin [13]. In the current study, we aimed to investigate the further molecular mechanism of deer antler aqueous extract on regulation of the hair cycle and hair follicle cell proliferation.

## 2. Materials and Method

### 2.1. Preparation of Deer Antler Extract

Four-year-old male red deer (*Cervus elaphus*), at the early, fast-growing stage of antler development, were obtained from a local deer farm. Analgesia was achieved by intramuscular injection of 20 mg/mL of xylazine hydrochloride at 1 mL/kg of body weight. The antlers were removed by cutting the proximal region using a surgical handsaw [14] and then cut into four sections (tip, upper, mid, and base). Once dissected, tissues from each region were sliced into 0.3 mm pieces using a trephine, snap-frozen in liquid nitrogen within 20 min of removal, and stored at −70°C until further analysis. The tips of antler tissues were homogenized in a Waring blender and then grounded into powder under liquid nitrogen. We used PBS buffer solution and water to extract deer antler, respectively, with different times. Compared to PBS buffer, water was able to extract more lower molecular protein which can be absorbed well, so water was deemed to be a superior extract solution and more suitable for subsequent experiments. To prepare 4°C aqueous extract, 5 g of deer antler powder was extracted in 50 mL of deionized water at 4°C for 1 h. The mixture was centrifuged at 10,000× g for 10 min at 4°C, and then the supernatant was filter-sterilized. Another 5 g deer antler powder was boiled in a water bath in 50 mL deionized water for 1 h to prepare the 100°C extract and the volume was filled up to 50 mL after boiling (since a little liquid was evaporated when boiling). The mixture was centrifuged at 10,000× g for 10 min at 4°C, and then the supernatant was filter-sterilized. Both of the two groups of total crude extract concentration were 100 mg/mL, stored at −70°C and thawed immediately prior to use.

### 2.2. Animal Treatment

Healthy C57BL/6 male mice purchased from Danhan Biolink Inc. (Korea) were used in all experiments and fed standard mouse chow and water. Animals were housed in polypropylene cages and maintained under standard conditions of a 12 h light/dark cycle, a temperature of 23 ± 2°C, and 35–60% humidity. All mice were kept in quarantine for one week prior to experimentation. Care and handling of all animals were done in accordance with guidelines for the care and use of laboratory animals of Chungnam National University. All protocols were approved and monitored by the Ethics Committee of Animal Experiments at Chungnam National University.

Mice in all treatment groups were morphologically preselected to be in the telogen phase (62 days) of the hair growth cycle. To achieve synchronized anagen development over the entire dorsal skin area, all mice were depilated with animal clippers and then treated with a commercial depilatory cream (Bikiro cream, Tai Guk Pharm. Co. Ltd., South Korea). The study included three groups of mice with their backs shaved (*n* = 9): group I was injected with 0.1 mL water only and served as control, group II was injected with 0.1 mL (100 mg/mL) 100°C deer antler aqueous extract (AAE), and group III was injected with 0.1 mL (100 mg/mL) 4°C AAE. All mice were subcutaneously injected into the dorsal skin for 10 consecutive days, started immediately after they were shaved, and sacrificed on the 10th day after depilation. Full-thickness skin samples were harvested, fixed in 4% paraformaldehyde (PFA), dehydrated, and embedded in paraffin for section. The remaining skin samples were stored at −70°C for future experiments.

### 2.3. Histological Studies

A 12 cm^2^ patch of dorsal skin was excised between fore and hind legs after injection with AAE at indicated times. Dorsal skin was fixed in 4% paraformaldehyde (PFA) and then embedded in paraffin. Consecutive 3 to 4 *μ*m thick longitudinal and transverse sections were then prepared. Sections were stained with hematoxylin and eosin (HE). Digital photomicrographs were taken from representative areas at the indicated magnification. Follicles were counted manually in the dermis and the subcutaneous layer by a blinded observer at a fixed magnification. Skin thickness from the epidermis to the panniculus carnosus was measured.

### 2.4. Statistical Analysis

In HE-stained sections, hair growth data are expressed as mean follicle length ± standard errors of at least three independent experiments performed in triplicate. Regrown hairs were plucked from representative areas in shaved parts of sacrificed mice on the 10th day. Average hair length from 30 hairs per mouse was calculated. Digital photomicrographs were taken from representative areas of slides at a fixed magnification of 100x. All images were cropped in a fixed area with a width of 600 *μ*m. Hair follicles in deep subcutaneous tissue (*n* = 30/mouse) were then manually counted for each group.

Student's *t*-test was used to assess differences between the values from the various experimental and control groups. Statistical significance was defined as *P* value < 0.05.

### 2.5. BrdU Labeling

Epithelial cell proliferation was measured by intraperitoneal (i.p.) injection of BrdU (130 *μ*g/g of body weight, Sigma, Korea) 48 h before sacrifice. Dorsal skin was excised and subjected to immunohistochemistry for BrdU. The number of BrdU-positive cells and the total number of cells were determined per 100 *μ*m of interfollicular epithelium in each section. For BrdU immunohistochemistry, paraffin sections were blocked with 1% bovine serum albumin (BSA) and then incubated with BrdU primary antibody for at least 1 h. Slides were washed three times with PBS and then incubated with conjugated secondary antibody for 20 min (Invitrogen, Carlsbad, CA). Finally, the reaction was visualized with diaminobenzidine as a chromogenic substrate for peroxidase. Digital photomicrographs were taken from representative areas of slides at a fixed magnification of 100x by light microscopy. All images were cropped in a fixed area with a width of 600 *μ*m. BrdU-positive cells in the hair matrix area and outer root sheath were then manually counted in a fixed area with a width of 300 *μ*m. In the meantime, we counted the S-phase cell in anagen in different parts of hair follicle as compared: matrix part, hair follicle papilla and hair shaft part. We took the picture at the magnification, of 100x and 400x. Nine sections were reduplicated in each group. Student's *t*-test was used to assess the differences between groups. All statistical analyses were performed in SPSS, version 18 (SPSS Inc., Chicago, IL, USA).

## 3. Results

### 3.1. Histological and Quantitative Morphologic Analysis of Hair Follicles

**Figure 1:**
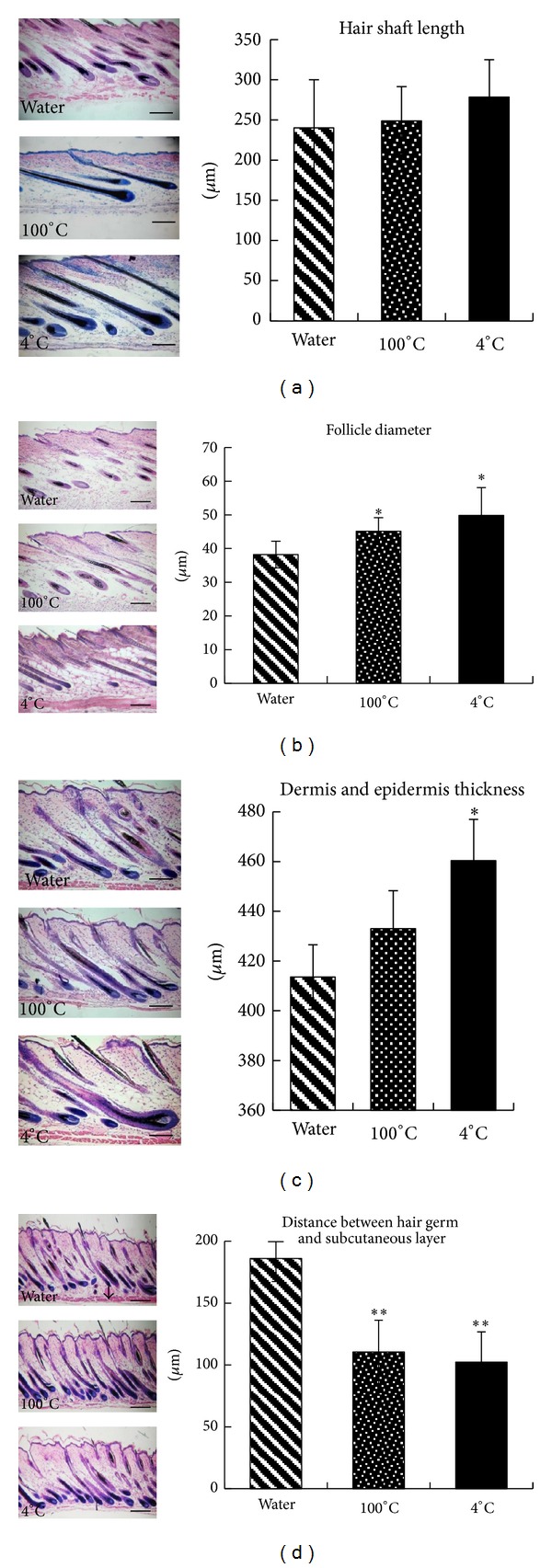
HE staining and quantitative morphologic analysis of hair follicles. HE-stained paraffin sections of dorsal skin of control (water) and 100°C or 4°C deer antler extract-treated mice were examined at 100x magnification. (a) Quantitative analysis of the hair shaft length. (b) Hair follicle diameter of the different groups. (c) Thickness of the dermis and epidermis in the different groups. (d) Distance between the hair germ and the subcutaneous layer in the different groups. ((a)–(d) left part) Sections of the back skins were stained, and representative photomicrographs of skin sections are shown. Bars are 100 *μ*m. Values are mean ± standard deviation (SD) (*n* = 10/mouse; *P < 0.05 and **P < 0.01 compared to control). Nine sections were reduplicated in each group.

The hair shaft length in the 4°C group was significant longer than control group, while 100°C group showed modest result (Figure 1(a)). Compared with control group, hair follicle diameter measurement in both 4°C group and 100°C group showed significant difference (Figure 1(b)).

The distance between the hair germ and the subcutaneous layer can be used to determine the phase of hair growth [15]. Based on these measurements, we found that the bulbs of the hair follicles in anagen were more closely associated with adipose tissue in the AAE groups, indicating that follicles penetrated deeper into the subcutaneous tissue during anagen phase in the AAE groups (Figure 1(c)). A significant difference between the 4°C group and the control group was also observed.

During the anagen phase, the hair bulb moved deeper into the dermis and reached its deepest position within the subcutaneous fat. Therefore, the distance between the hair germ and the subcutaneous layer was the shortest during this phase. In the current study, the distance in both AAE groups (4°C and 100°C) was significantly shorter than in the control group (Figure 1(d)). Moreover, the number of hairs in both AAE groups was greater than in the control group, as shown in the histology photograph in Figure 1(d).

### 3.2. Immunohistochemical Analysis of BrdU Retention in Matrix and Outer Root Sheath

**Figure 2:**
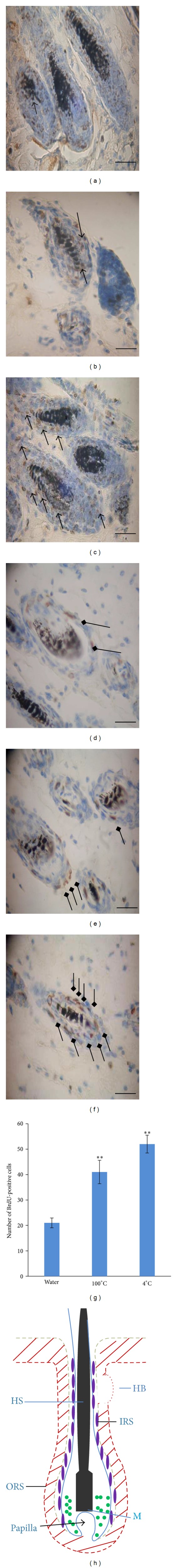
Immunohistochemical analysis of BrdU retention in matrix and outer root sheath region. BrdU staining in hair follicles from 7-week-old C57BL/6 mice treated with water, 100°C and 4°C AAE at 100x magnification. ((a)–(c)) Vertical section BrdU incorporation cell in matrix part in the water, 100°C AAE and 4°C AAE group (open arrows indicated BrdU-positive cells). ((d)–(f)) Transverse sections BrdU incorporation cell in outer root sheath part in the water, 100°C AAE and 4°C AAE groups (diamond arrows indicated BrdU-positive cells). (g) Number of BrdU-positive cell in a fixed area with a width of 300 *μ*m. (h) Schematic diagram of a hair follicle showing key components. Abbreviations: HS: hair shaft; M: hair matrix; IRS: inner root sheath; ORS: outer root sheath. Scale bars are 100 *μ*m.

Since hair follicle formation during anagen requires cell proliferation in the matrix, BrdU incorporation was used to investigate whether the increase in hair shaft growth was due to hyperproliferation in the matrix region. Results indicated that both AAE groups dramatically increased cell proliferation in the hair matrix and bulge regions during anagen phase (Figures 2(a)–2(f)). Moreover, when cell proliferation in a section of the hair follicle was quantified at day 10, an increase in the number of positive cells was observed (Figure 2(g)). Both transverse and vertical sections showed that BrdU incorporation in AAE groups was significantly increased compared to control group (Figures 2(a)–2(f)). The BrdU labeling index in AAE groups was significantly higher than in vehicle control, especially in the hair matrix (Figures 2(a)–2(f), open arrows) and the outer root sheath (Figures 2(a)–2(f), diamond arrows), which contained a subpopulation of outer root sheath cells located in the middle portion of a follicle at the arrector pili muscle attachment site (the niche shown in Figure 2(h)). Data shown represents the mean ± standard deviation (SD) of three independent experiments. One-way ANOVA was used to compare groups, followed by Dunnett's *t*-test.

### 3.3. BrdU Incorporation during S-Phase of Anagen

**Figure 3:**
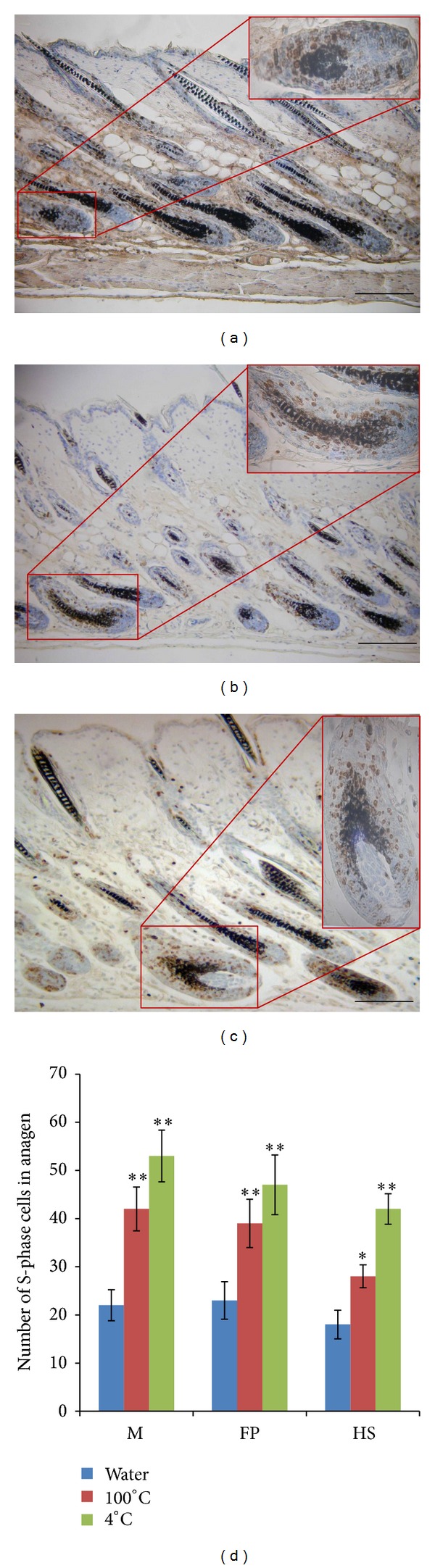
Immunohistochemical staining of BrdU incorporation in S-phase in anagen. ((a)–(c)) Paraffin sections from a 7-week-old C57BL/6 mouse injected with bromodeoxyuridine 48 h before necropsy, at magnification 100x. LRC could be seen in the matrix and the follicle papilla after labeling during the onset of anagen. Note the high proliferation rate (number of brown nuclei) in AAE-treated animals ((b) and (c)) compared to the control (a), with (d) showing the number of S-phase cells during anagen phase in different hair follicle regions. M: matrix, FP: follicle papilla, and HS: hair shaft. Scale bars are 100 *μ*m.

BrdU immunodetection was used to quantify proliferating cells in hair follicles during the growth phase (S-phase). BrdU-positive cells were observed in the hair follicle outer root sheath, matrix cells of the hair shaft, and the follicle papilla. After being injected with AAE (4°C and 100°C), the number of labeled cells which were in S-phase increased dramatically in the mouse matrix region, hair shaft, and the follicle papilla part. (Figure 3).

## 4. Discussion

In the current study, the potential role of deer antler extract (AAE) in promotion of hair growth via regulation of cell proliferation in the hair follicle region was investigated.

Histological analysis revealed that mice hair follicles treated with AAE were dramatically stronger and contained more proliferating cells than vehicle control, with longer and sturdier hair, as well as more hairs in the flourishing growth phase. The effect of stimulating hair growth was superior in the 4°C AAE group than in the 100°C AAE group. Althought here was, no significant effect on elongation of hair length or increasing hair follicle diameter, the hair anagen phase was more obviously extended. Mechanisms that subsequently observed stimulation of hair growth remained to be determined. In traditional Chinese medicine, deer antler was sliced very thinly and typically boiled for several hours to release the gelatin (protein components) [16]. Therefore, two extraction temperatures were tested: 100°C (based on traditional usage) and 4°C (based on the biological optimum). AAE contains many cytokines, dehydrogenases, alkaline phosphates, and many essential amino acids [17]. A high temperature, such as 100°C, was likely to result in protein denaturation [18]. As such, enzymatic activity in 100°C AAE was likely less than in 4°C AAE. In this study, BrdU, a synthetic nucleoside analogue of thymidine that is incorporated into newly synthesized DNA of replicating cells (S-phase of the cell cycle), was used to detect proliferating cells in living tissues [19, 20]. The BrdU labeling index in both AAE groups was significantly higher than the vehicle group, especially in the hair matrix and outer root sheath part (Figures 2(a)–2(f)). Hair follicle stem cells are thought to be slow cycling cells [21], which, in addition to high proliferative potential, is a characteristic feature of stem cells [22]. Cells stained with BrdU are referred to as label-retaining cells (LRC) [23, 24].

Presumably because stem cells are highly proliferative during development, they can be labeled with BrdU during the neonatal period. Studies in mice clearly demonstrate that LRC are present in both the epidermis and the hair follicle bulge [25]. In the hair follicle, Rizvi and Wong [26] determined that stem cells are located in the outer root sheath and the hair matrix around the dermal papilla [27], which is consistent with data from the current study.

Several possible explanations as to why AAE may stimulate cell proliferation in the follicle region have been proposed. For instance, AAE contains high levels of insulin-like growth factor (IGF)-1 especially in tip part which were reported in our previous study [6]; we isolated the IGF-1 from fresh antler tissue and we purified of protein [28]. IGF-1 is a low molecular weight protein of roughly 7 kDa that may stimulate hair growth, as it is critically involved in regulating cellular proliferation and migration during the development and growth cycles of hair follicles [29]. Suttie et al. [30] showed that plasma levels of IGF-I, but not IGF-II, were significantly elevated during velvet antler growth in red deer. Furthermore, velvet antler growth rate has been strongly correlated with increasing levels of IGF-I in plasma. Lastly, IGF-I and II are polypeptide hormones known to stimulate cell proliferation [31], as well as DNA synthesis and matrix production [27].

Vimentin, which is also one of the most abundant proteins in deer antler [3], is recently used to inhibit hair loss through the functions of promoting the proliferation of dermal papilla cells and increasing cell migration and growth factor expression [32].

In 2002, Yegorova [33] reported that deer antler may inhibit 5*α*-reductase, thus reducing undesirable levels of dihydrotestosterone (DHT), maintaining testosterone levels and avoiding production of undesirable metabolites [34]. DHT is the primary contributing factor in male pattern baldness, which is referred to as androgenetic alopecia. Androgenetic alopecia is a dihydrotestosterone- (DHT-) mediated process, characterized by continuous miniaturization of androgen-reactive hair follicles and perifollicular fibrosis of follicular units, which are detected by histological examination [35]. Retention of late anagen follicles as well as increasing follicular length and prevention of their miniaturization might therefore be attributed to 5*α*-reductase inhibitory activity. Male balding involves a change in the hair cycle and size of hair follicles. The reduction in anagen duration predominates so that the overall effect is a shortening of the hair cycle.

Velvet antler is living tissue that grows at a rate of up to 2 cm/day in some species. Cartilage, bone, and supporting tissues, such as nerves, blood vessels, and hair follicles, of the antler also exhibit accelerated growth. It is believed that factors that drive rapid regeneration of velvet antler may explain the observed health benefits of traditional Chinese medicines prepared from velvet deer antler. A wide variety of growth factors can be found in velvet and may be associated with its growth-promoting activity. Some key components in velvet deer antler include lysophosphatidylcholine, which possesses hypotensive activity [36], improving circulation and regulating blood flow, which is essential for stimulation of the scalp and hair growth.

Identification of individual active components of deer antler will require further research. In the current study, it was demonstrated that selecting functional factors in deer antler may play an important role in regulating the hair cycle and promoting hair cell proliferation in rodents. Deer antler may therefore provide a potential new treatment to promote hair regrowth.

## 5. Conclusions

These results indicate that deer antler aqueous extract promotes hair growth by extending the anagen phase and regulating cell proliferation in the hair follicle region. We suggest that some unidentified functional factors in deer antler, such as IGF-1, may play a role in regulating the hair cycle and follicle cell proliferation in rodents.
